# Supplementary material for: A comparison of super‐resolution microscopy techniques for imaging tightly packed microcolonies of an obligate intracellular bacterium
Source: J Microsc. 2024 Dec 9;301(2):187–205. doi: 10.1111/jmi.13376 (PMC12884446; doi:10.1111/jmi.13376)
Supplement: Supplementary file 1 — Supporting Information [file JMI-301-187-s002.docx]

**
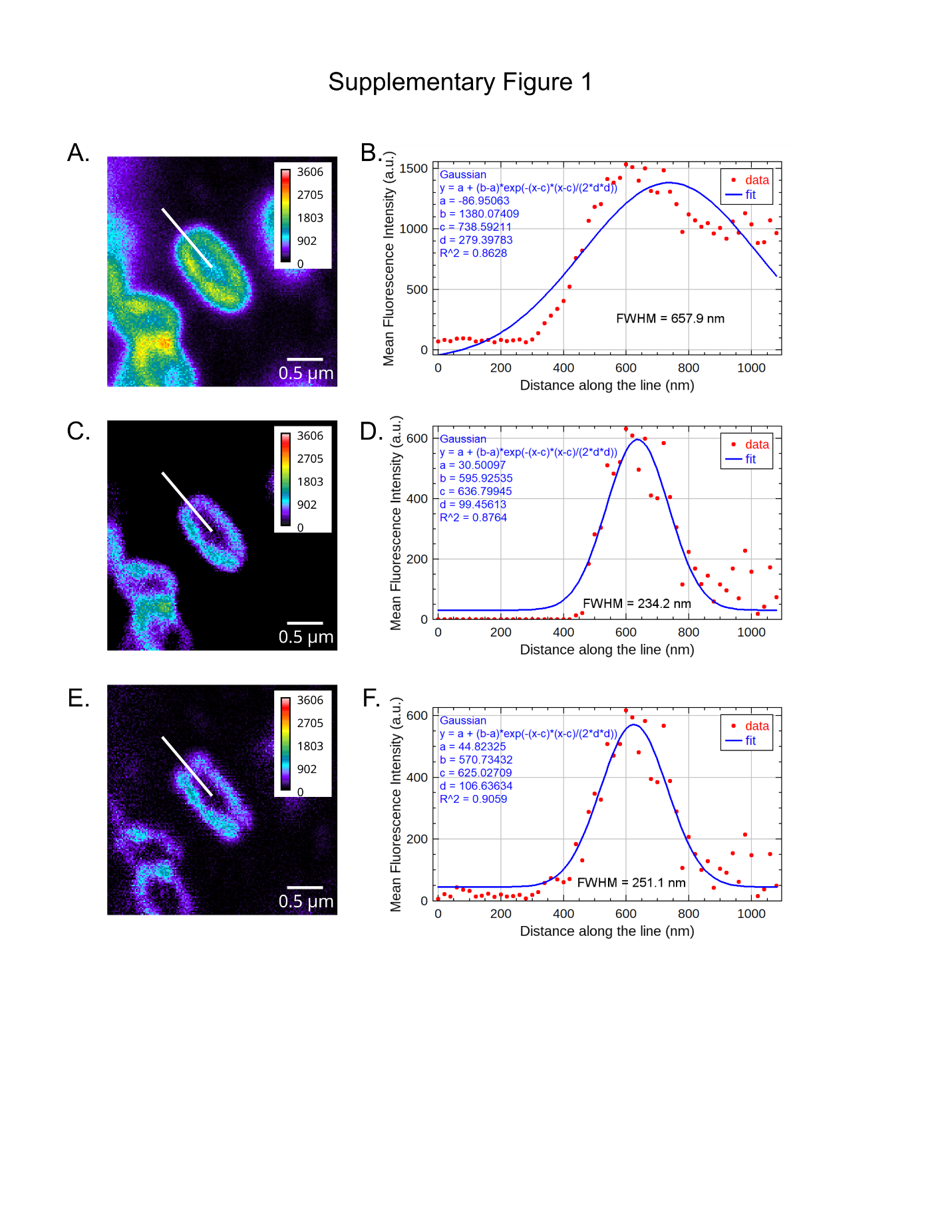
**

**Supplementary Figure 1.** High Ot cytoplasmic background in standard confocal image resulted in poor curve fitting, and enlarged,inaccurate FWHM measurements.

(A) Cropped confocal image of Ot bacteria (AF 488 labelling). Confocal images exhibited high cytoplasmic background inside the Ot bacteria, 20.4 higher than the host-cell cytoplasmic background outside the bacteria, and with a ScaA signal only 1.37 times higher than the Ot cytoplasmic background.

(B) FWHM plot of line ROI from (A). The high Ot cytoplasmic background and asymmetric background on either side of the ScaA signal made Gaussian fitting challenging, exaggerated the fitting and resulting in an inaccurate FWHM measurement. Gaussian fitting formula, parameter values (a, b, c and d) and goodness of fit (R^2^) values are on the graph.

(C) Cropped confocal image of Ot bacteria (AF 488 labelling) after applying a constant background subtraction of 900 A.U.

(D) FWHM plot of line ROI from (C). The constant background subtraction truncated the signal intensity and led to an unrealistically low FWHM measurement.

(E) Cropped confocal image of Ot bacteria (AF 488 labelling) after applying rolling ball (radius = 11 pixels) background subtraction.

(F) FWHM plot of line ROI from (E). While the rolling ball background subtraction produced a Gaussian fit more reflective of theoretical confocal performance, it carries the risk of yielding inaccurate and unrepresentative FWHM measurement and optimising rolling ball radius settings was required for each image.

**
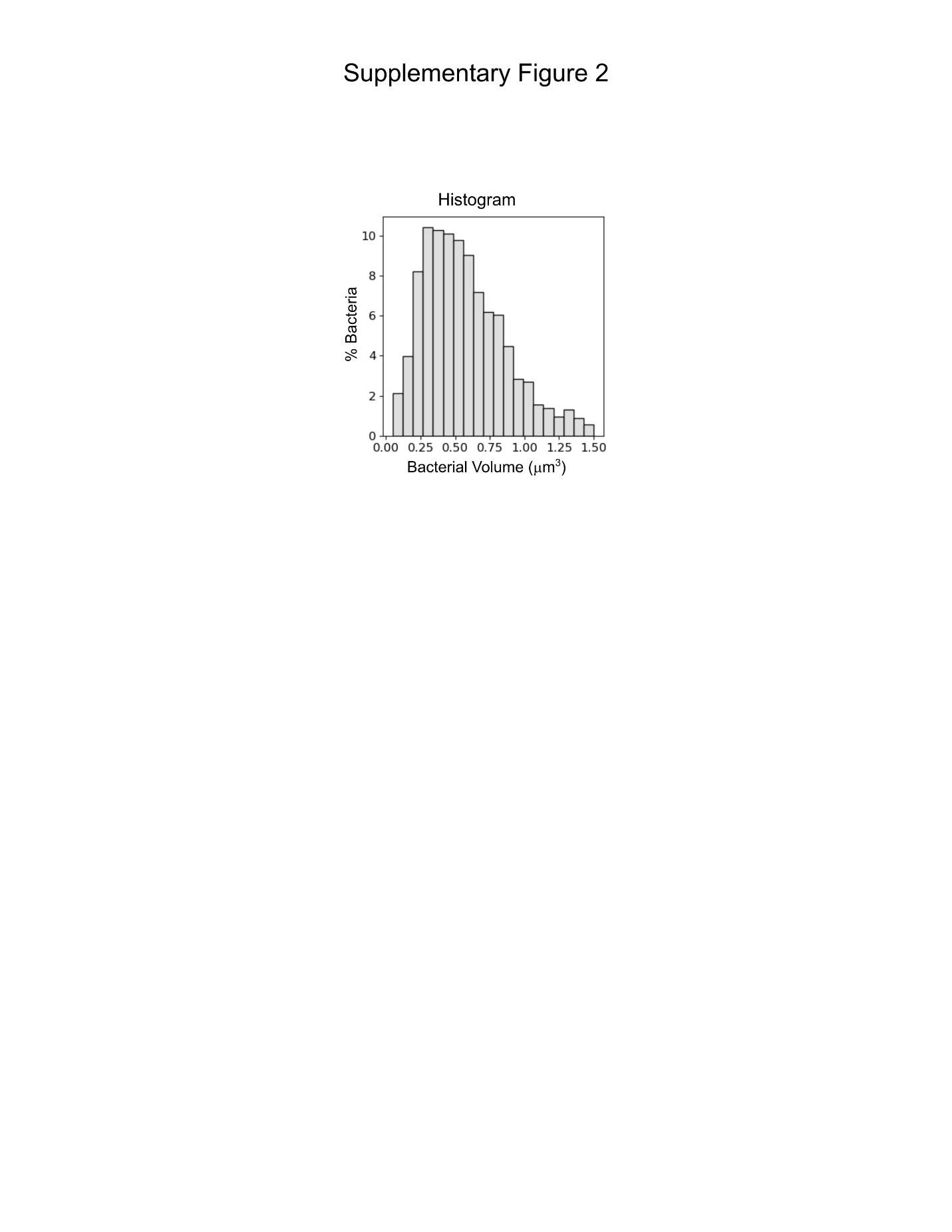
**

**Supplementary Figure 2.** Bacterial volume distribution for bacteria aggregates in HeLa cells imaged using 3D STED. In total, n= 1262 (3D STED) bacteria were analyzed for histogram generation.
